# Protective Effect of Intestinal Helminthiasis Against Tuberculosis Progression Is Abrogated by Intermittent Food Deprivation

**DOI:** 10.3389/fimmu.2021.627638

**Published:** 2021-04-14

**Authors:** Cristina Garrido-Amaro, Paula Cardona, Diana Gassó, Lilibeth Arias, Roser Velarde, Asta Tvarijonativiciute, Emmanuel Serrano, Pere-Joan Cardona

**Affiliations:** ^1^ Wildlife Ecology & Health Group (WE&H) and Servei d’Ecopatologia de Fauna Salvatge (SEFaS), Universitat Autònoma de Barcelona (UAB), Bellaterra, Spain; ^2^ Unitat de Tuberculosi Experimental, Institut Germans Trias i Pujol, UAB, Badalona, Spain; ^3^ Centro de Investigación Biomédica en Red de Enfermedades Respiratorias (CIBERES), Madrid, Spain; ^4^ Departament of Animal Science, Agrifood, Forestry and Veterinary Campus, University of Lleida, Lleida, Spain; ^5^ Interdisciplinary Laboratory of Clinical Analysis Interlab-UMU, Regional Campus of International Excellence Campus Mare Nostrum, Universidad de Murcia, Murcia, Spain

**Keywords:** co-infection, tuberculosis, *Trichuris muris*, *Heligmosomoides polygyrus*, environmental mycobacteria *Mycobacterium manresensis*, fasting, C3HeB/FeJ

## Abstract

**Background:**

Tuberculosis (TB) is still a major challenge for humankind. Because regions with the highest incidence also have a high prevalence of helminthiasis and nutritional scarcity, we wanted to understand the impact of these on TB progression.

**Methods:**

We have developed an experimental murine model for active TB in C3HeB/FeJ, coinfected with *Trichuris muris* and *Heligmosomoides polygyrus* nematodes, and exposed to an environmental mycobacterium (*M. manresensis*) and intermittent fasting. Cause-effect relationships among these factors were explored with Partial Least Squares Path modelling (PLSPM).

**Results:**

Previous parasitization had a major anti-inflammatory effect and reduced systemic levels of ADA, haptoglobin, local pulmonary levels of IL-1β, IL-6, TNF-α, CXCL-1, CXCL-5 and IL-10. Oral administration of heat-killed *M. manresensis* resulted in a similar outcome. Both interventions diminished pulmonary pathology and bacillary load, but intermittent food deprivation reduced this protective effect increasing stress and inflammation. The PLSPM revealed nematodes might have protective effects against TB progression.

**Conclusions:**

Significantly higher cortisol levels in food-deprivation groups showed it is a stressful condition, which might explain its deleterious effect. This highlights the impact of food security on TB eradication policies and the need to prioritize food supply over deworming activities.

## Introduction

Tuberculosis (TB) is an infectious disease caused by *Mycobacterium tuberculosis* (Mtb) and is a major global health problem. In 2018, 10 million people fell ill with TB and it caused 1.4 million deaths worldwide (1). Most TB infection occurs in regions where parasitization by helminths is highly prevalent (2).

Soil-transmitted intestinal parasites are also a global health problem, with *Ascaris lumbricoides*, *Trichuris trichiura* and *Necator americanus* infecting an estimated 804, 477 and 472 million people respectively worldwide (3). The response to helminth infection can be summarized as causing a bias toward Th2 and an increased Treg response, which modulates both Th1 and Th2, a bias that may be transmitted to infants *in utero* (4). Due to the importance of Th1 responses in controlling Mtb infection, it is constantly questioned whether to advocate for a deworming policy or not as a coincidental factor to improve TB vaccination programs. Equally, it is presumed that people in these regions have a high level of contact with environmental mycobacteria, which has a clear impact in the immune response against Mtb. In fact, it is one of the reasons believed to explain the failure of the protection induced by BCG vaccination in several settings (5), either because exposure to environmental mycobacteria provides some protective immunity to TB (6, 7) or because it blocks the replication of BCG and thus its protective effect (7, 8).

Additionally, food insecurity is also common in these regions (9). Body mass index (BMI) below 18.5 is a well-known risk factor for TB progression (10) due to the reduction of essential nutrients required to build an appropriate immune response. However, little is known about the effect of intermittent food deprivation, as occurs in a state of food insecurity, and the impact of the stress it causes, even when an overall malnutrition has not been reached.

Surprisingly, our data shows a protective effect of intestinal helminthiasis related to its anti-inflammatory properties, which is abrogated by intermittent food deprivation, thus reinforcing the importance of food security in any TB eradication program.

## Materials and Methods

### Animals

A total of 24 female and 36 male C3HeB/FeJ specific-pathogen-free mice (8–10 weeks old) were obtained from Germans Trias i Pujol Research Institute stock. All procedures were conducted in a BSL-3 facility, according to protocol DMAH6119. This was reviewed by the Animal Experimentation Ethics Committee of the Hospital Universitari Germans Trias i Pujol (registered as B9900005) and approved by the Departament d’Agricultura, Ramaderia, Pesca, Alimentació i Medi Natural of the Catalan Regional Government, according to current national and European Union legislation regarding the protection of experimental animals. Mice were supervised daily following a strict monitoring protocol to ensure animal welfare and euthanized, if required, by cervical dislocation after anesthesia with isoflurane.

### Experimental Design

**Figure 1:**
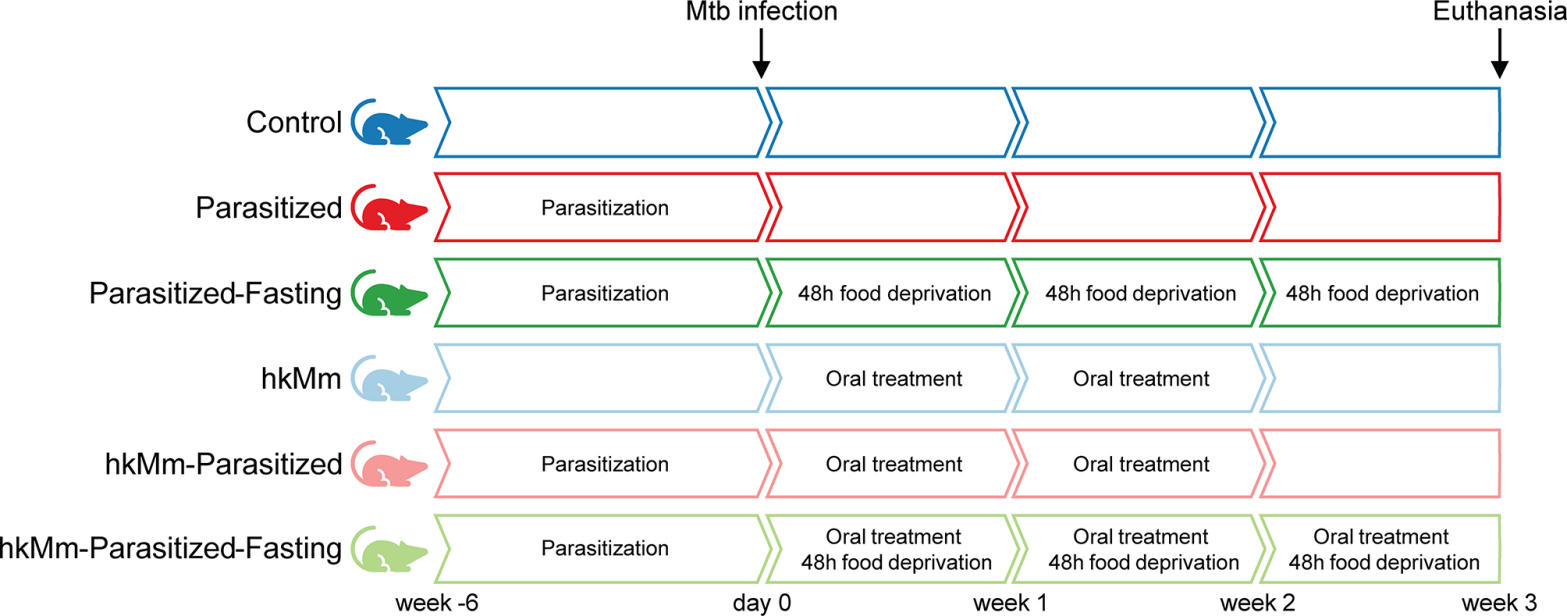
Experimental design. Six groups of 10 animals were used to evaluate the influence of intestine parasitization, intermittent fasting and oral exposure to environmental mycobacteria in an active TB model.

Animals were divided into 6 experimental groups (**Figure 1**), 10 mice per group evenly matched in sex and weight.

Four groups were orally infected by gavage, twice, with 0.2 mL of a solution containing 30 *Trichuris muris* eggs and 200 *Heligmosomoides polygyrus* larvae as previously done (11, 12). Once the parasitization had been confirmed by coprologic examination, 6 weeks after parasite infection, all animals were infected with 2×10^4^ CFU (colony-forming units) of *M. tuberculosis* H37Rv Pasteur strain *via* the caudal vein (13).

Environmental mycobacterial treatment started one day after Mtb infection. Mice received oral treatment with 10^5^ CFU of heat-killed *M. manresensis* (hkMm) (14) in a galenic formulation with mannitol. Control groups received mannitol in the same concentration. A total of 7 doses were administered, every other day, for 14 days.

Two groups of animals were submitted to intermittent fasting throughout the experiment, with a weekly regime of 5 days of normal diet and 2 days of food deprivation (15). Otherwise, mice received food and water *ad libitum*.

Animal weight was recorded every 2-3 days. Fecal samples were obtained from each cage weekly to quantify parasite eggs. After 3 weeks from Mtb infection, mice were anesthetized with isoflurane inhalation and blood samples were obtained through cardiac puncture. They were then euthanized by cervical dislocation. Lungs, spleen, liver, kidneys and intestines were retrieved. Kidneys and liver were weighed in a precision balance with 0.0001 g accuracy.

### Bacillary Load (BL)

Spleen and left lobe lung samples from each animal were collected, homogenized and 10-fold serial dilutions were made. 100 µL of these dilutions were plated on nutrient Middlebrook 7H11 agar (BD Diagnostics, Sparks, USA). Visible CFU were counted after incubation for 28 days at 37°C. Data was analyzed as CFU per mL.

### Lung Pathology

Right caudal lung lobe samples were fixed in 10% buffered formalin, embedded in paraffin and 5-μm sections stained with hematoxylin-eosin for microscopic observation. Two paraffin blocks per group were obtained, each containing samples from 5 animals. Four recuts of every block were used to determine the damaged area as a percentage of total lung area. This analysis was done using the NISElements D version 3.0x software package (Nikon Instruments Inc., Tokyo, Japan).

### Oxidative Stress, Inflammatory Mediators and Cortisol in Serum

Blood samples were extracted in tubes containing clotting activator (Sarstedt, Nümbrecht, Germany). After centrifugation, sera were obtained and stored at -20°C until analysis.

Total antioxidant capacity was determined by four methods: trolox equivalent antioxidant capacity (TEAC1 and TEAC2), ferric reducing ability of plasma (FRAP) and cupric reducing antioxidant capacity (CUPRAC), as previously reported (16). Thiol was determined using method reported by Jocelyn (17). PON1 activity was analyzed following previously described method (18) and advanced oxidation protein product (AOPP) concentrations as described by Witko-Sarsat (19). All procedures were performed in an automated biochemistry analyzer (Olympus AU600, Beckman Coulter, Brea, USA). Reactive oxygen species (ROS) were estimated by luminol-mediated chemiluminescence assay (20) using a microplate reader (Victor 2 1420 Multilabel Counter; PerkinElmer, Finland).

Total adenosine deaminase (ADA), albumin and haptoglobin were measured using commercially available kits (Diazyme Laboratories, Poway, CA, USA; Beckman Coulter, USA and Tridelta Development, Ireland, respectively) in an automated biochemistry analyzer (Olympus AU600, Beckman Coulter, Brea, USA). Cortisol was analyzed following Arantes-Rodrigues et al. (21) and using a commercially available solid-phase, competitive chemiluminescence enzyme immunoassay (COR Cortisol, Siemens Health Diagnostics, Deerfield, IL, USA) in the automatic analyzer (Immulite 1000, Siemens HealthcareDiagnostic, Deerfiels, IL, USA).

### Lung Immune Response

A cytokine profile study was performed in lung homogenates from right cranial and middle lobes. The following cytokines were measured by Luminex xMAP^®^ technology: IFN-γ, TNF-α, IL-1β, IL-6, IL-10, IL-12(p40), IL-13, IL-17, CXCL-1 and CXCL-5. Results are expressed as pg per mL of homogenate. The assay was performed with the MILLIPLEX^®^ MAP kit (EMD Millipore Corporation, Billerica, MA, USA) following the manufacturer’s instructions and analyzed with xPONENT Software (Luminex Corporation, Austin, TX, USA).

### Data Analysis

#### Multiple Comparisons

The scores of the first dimension (PC1) of a principal component analysis (PCA) on liver and kidneys weights were used as proxy for body condition in mice. Likewise, the PC1 of a PCA for the antioxidant (TEAC1, TEAC2, FRAP, CUPRAC, Thiol and PON1) and oxidant (AOPP and ROS) biomarkers was used as proxy for the oxidative stress profile. The same approach was followed for lungs immune response (analytes detailed in section 2.6). Differences in the PCA scores among experimental groups were explored with ANOVA and a Holm-Sidak post-hoc multiple comparison tests (22, 23).

On the other hand, a Mann-Whitney test was used to compare damage area and BL among experimental groups. The same statistical test was used to compare differences among treatments in concentration of oxidative stress mediators in serum and cytokines/chemokines in lungs.

PCA was performed using the package “FactomineR” version 2.0 (24) of the statistical software R 3.6.2 version (25). Graphpad Prism (GraphPad software v7.0, La Jolla, California, USA) was used for graphics and simple statistics, with differences of p<0.05 being considered statistically significant. Statistically significant differences are summarized as follows: *p<0.05, **p<0.01, ***p<0.001.

#### PLS Path Modeling

We used Partial Least Square Path Modelling (PLS-PM) (26–29) to explore the association of intermittent fasting, helminth infection and hkMm with Mtb load. Briefly, this approach quantifies the network relationship between a set of unobservable latent variables (LV) and a set of parameters directly measured (manifest variables or MV). The LVs are conceptual variables defined by one or several MVs and organized in a network of relationships where the connections among LVs are assumed to represent a cause-effect process called inner model. The links among LV are quantified through path coefficients while the links between LV and MV are quantified through weights (30).

Our PLS-PM included 28 MVs organized into 10 LVs, described in **Supplementary Table 1**. The initial inner PLS-PM model is shown in **Supplementary Figure 1**. The assumed direct effects for the model structure are detailed in **Supplementary Table 2**. Even though PLS-PM is distribution free, some variables (IFN-γ, IL-10, IL-12, IL-17, parasite burden and BL) were log-transformed to improve the model fit. After fitting the first model including all the variables, we performed a simplification removing those MVs uncorrelated with their own LVs (**Supplementary Table 3**), following Sanchez (26). Finally, we also estimated the partial contribution of each LV to the final PLS-PM as well as the goodness of fit [GOF, (31)]. PLS-PM analysis was performed using the package ‘plspm’ version 0.4.9 (26) of the statistical software R 3.6.2 version (25).

## Results

### Intermittent Fasting Had a Low Impact on Body Condition

**Figure 2:**
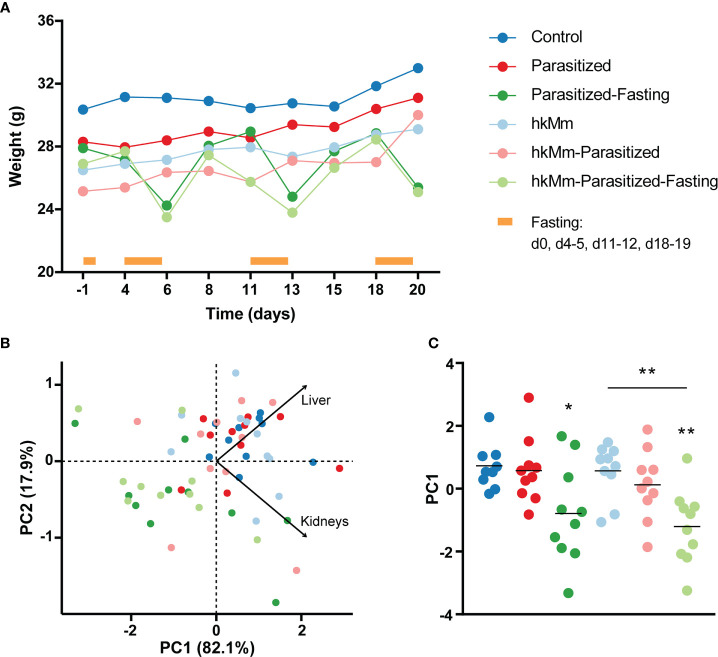
Change of animals’ body condition. **(A)** Median weight of mice as infection progresses, infection day being time 0. **(B)** PCA based on liver and kidneys weight at end-point time. **(C)** PC1 scores, each circle represents an animal and lines are means; ANOVA *p*-value=0.0004, Holm-Sidak’s multiple comparisons test (*p < 0.05, **p < 0.01). hkMm, heat-killed *M. manresensis*.

Fasting mice reduced their body weight in a transitory manner (**Figure 2A**). Nonetheless, the effect of the intermittent fasting was evidenced in liver and kidney weight at the final endpoint. This data was used to perform a PCA, showing that both fasting groups had statistically significant lower scores of PC1 (**Figures 2B, C**).

Albumin was also measured in sera, but there were no statistically significant differences between groups (**Supplementary Figure 2**).

The number of eggs per gram of feces was determined weekly for samples from each cage. At the endpoint of the experiment, the total number of parasites was determined in the intestines of each animal. Neither measurement showed significant differences between groups (**Supplementary Figure 3**).

### Intestinal Parasites and Oral hkMm Reduced Lung BL and Pathology

**Figure 3:**
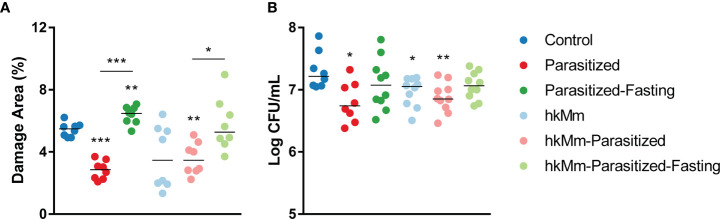
Damage area **(A)** and pulmonary bacillary load **(B)** at week 3 post-infection. Each circle represents a “recut” **(A)** or an animal **(B)** and lines show medians. Mann-Whitney test: *p<0.05, **p<0.01, ***p<0.001. hkMm, heat-killed *M. manresensis*.

The damaged area in lungs was reduced with parasitization and oral hkMm (**Figure 3A**). Representative lung sections of each group are shown in Supplementary **Figure 4**. In agreement with these results, the Mtb load in lungs was significantly reduced in the same groups (**Figure 3B**). This protective effect against the progression of active TB was not observed in fasting animals.

**Figure 4:**
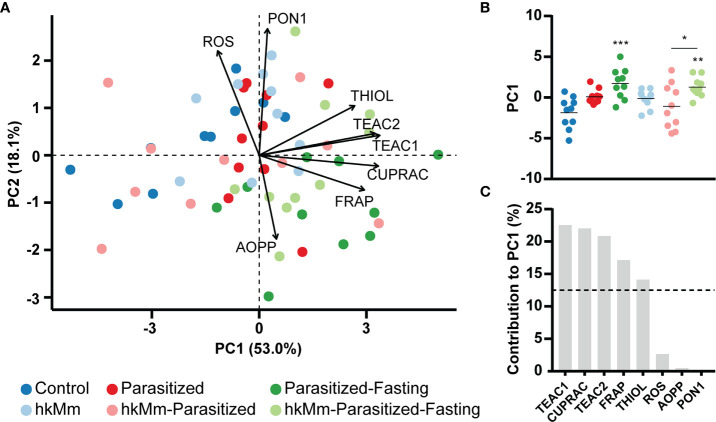
Oxidative stress analysis at week 3 post-infection. **(A)** PCA based on mediator concentration in serum. **(B)** PC1 scores, each circle represents an animal and lines are means; ANOVA *p*-value=0.0001, Holm-Sidak’s multiple comparisons test (*p < 0.05, **p < 0.01, ***p < 0.001). **(C)** Mediators contribution to PC1. hkMm, heat-killed *M. manresensis*; TEAC, trolox equivalent antioxidant capacity; CUPRAC, cupric reducing antioxidant capacity; FRAP, ferric reducing antioxidant power; PON1, paraoxonase 1; ROS, reactive oxygen species; AOPP, advanced oxidation protein products.

There were no differences between groups for BL in spleen (**Supplementary Figure 5**).

### Fasting Induced Antioxidant Activity

Oxidative stress was measured in different ways in sera (**Supplementary Figure 6**). When compared with control animals, parasitized mice had a significant increase in TEAC2, CUPRAC and FRAP. This was also the case in parasitized-fasting, hkMm and hkMm-parasitized-fasting groups. Fasting animals also showed increased TEAC1 and reduced PON1, regardless of the oral treatment, and increased thiol and reduced ROS when not receiving hkMm. The hkMm group was the only one that presented lower levels of AOPP than control mice. The hkMm-parasitized group had no statistically significant differences from controls.

These results were used to perform a PCA (**Figure 4A**). The first two components explained 71.1% of the variation in the data set. Fasting induced a significant increase in the PC1 score (**Figure 4B**), which was mainly explained by TEAC1, CUPRAC, TEAC2, FRAP and thiol variation (**Figure 4C**).

### Intestinal Parasites and Oral hkMm Reduced Inflammation

**Figure 5:**
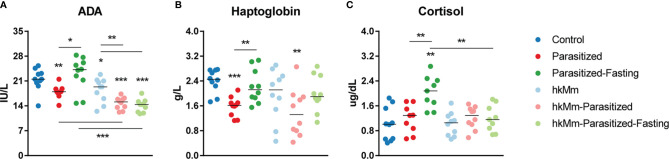
Adenosine deaminase (ADA) **(A)**, haptoglobin **(B)** and cortisol **(C)** levels in serum at week 3 post-infection. Each circle represents an animal and lines are medians, Mann-Whitney test (*p<0.05, **p<0.01, ***p<0.001). hkMm, heat-killed *M. manresensis*.

ADA, haptoglobin and cortisol were measured in sera (**Figure 5**). ADA was significantly reduced when compared with control animals in all groups except parasitized-fasting. The administration of hkMm in parasitized groups also showed a reduction in ADA levels when compared with their control counterparts. Parasitization induced a reduction of haptoglobin levels, but only under normal feeding conditions. Cortisol was significantly increased in parasitized-fasting animals.

Lung homogenates were used to quantify various cytokines and chemokines (**Supplementary Figure 7**). Parasitized animals showed a statistically significant reduction in TNF-α, IL-10, IL-1β, IL-6, CXCL-1 and CXCL-5 levels. Mice receiving oral hkMm had lower levels of TNF-α, IL-1β, IL-6, IL-17, CXCL-1 and CXCL-5. The levels of IL-13 were below the detection limit in all the samples (data not shown).

**Figure 6:**
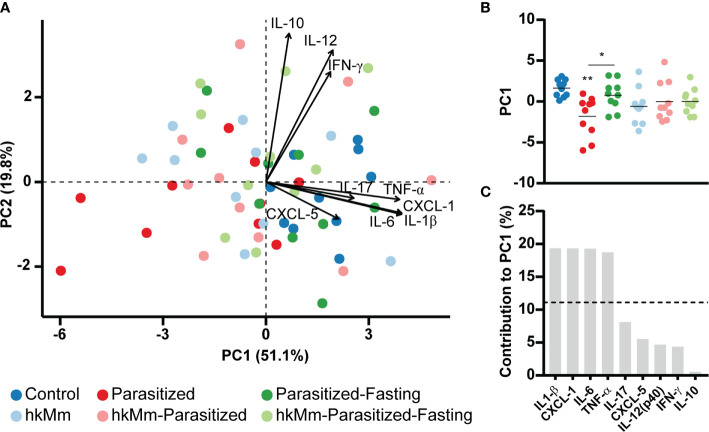
Immune mediator analysis at week 3 post-infection. **(A)** PCA based on cytokines concentration in lung homogenates. **(B)** PC1 scores, each circle represents an animal and lines are means; ANOVA *p*-value=0.0067, Holm-Sidak’s multiple comparisons test (*p < 0.05, **p < 0.01). **(C)** Mediators contribution to PC1. hkMm, heat-killed *M. manresensis*.

The PCA analysis of lung cytokines and chemokines showed the first two components accounted for 70.9% of the data set variation (**Figure 6A**). The PC1 score, based mainly on levels of IL-1β, CXCL-1, IL-6 and TNF-α, related to the induction of exudative lesions, was significantly reduced in the parasitized animals (**Figures 6B, C**).

### PLS Path modeling

According to the initial model (**Supplementary Table 4**), fasting increases helminth load, both fasting and helminths increase cortisol. Body condition is affected by helminths and fasting. Antioxidants appear increased in fasted mice and oxidation is reduced by the effect of antioxidants. Proliferative lesions were stimulated under food restriction. The bacillary load LV was negatively related to hkMm, helminths and cortisol, but positively related to exudative lesions and antioxidants. However, only exudative lesions were significantly related to BL in the lungs.

**Figure 7:**
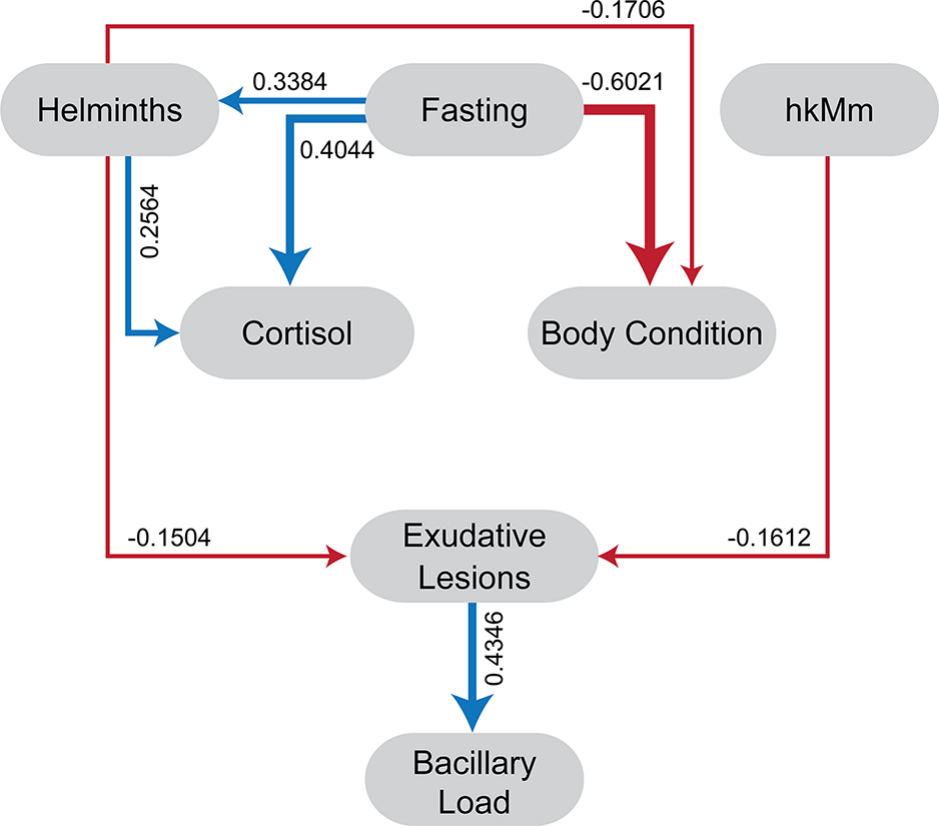
Refined partial least square path model. Red arrows indicate a negative relation among latent variables (path coefficient bellow 0) and blue arrows indicate a positive one (path coefficient over 0).

After removing the non-significant effects, antioxidant and oxidant LVs were isolated from the path, so these blocks were excluded from the refined model. The LV proliferative lesions was not related to bacillary load and hence also removed from the model. The final refined model shows both hkMm and helminths reduce exudative lesions that in turn are responsible for higher bacillary loads (**Figure 7**).

The goodness of fit for the model was 0.39 (39% of fit). The LV body condition had the greatest impact (46.1%) on the explained variability, followed by cortisol (29.9%) and bacillary load (18.8%).

## Discussion

In this work, we aimed to study the relation between three important factors affecting global health: tuberculosis, intestinal helminthiasis and food scarcity. Our data showed a protective effect of parasitization in the outcome of Mtb infection, which was abrogated by intermittent food deprivation.

Contrary to our findings, most data from cohort studies suggest an increased susceptibility to developing TB when coinfected. Helminthiasis infection has been linked to pulmonary TB in a cohort study that compared infected individuals with a control group (32). In another study, patients with TB and coinfected also presented a more advanced disease (33). Lower IFN-γ and higher IL-10 responses were found in coinfected TB patients after stimulation of whole-blood with mycobacterial antigens. Among pediatric household contacts of smear positive TB patients, a positive tuberculin skin test was significantly associated with coinfection (34). This was interpreted as helminth infection increasing the risk of acquiring latent tuberculosis infection (LTBI). This is in concordance with the improvement of Mtb-specific immune responses in PBMCs after deworming treatment in LTBI persons (35). All in all, these results are consistent with the attenuation of Th1 responses caused by helminths. However, even though the host’s defense against Mtb requires a Th1 response, it is not a direct correlate of protection (5). In fact, despite presenting higher levels of Tregs and Th2 cytokine response, helminth coinfection was related with a lower sputum smear-positivity (36). Recent data obtained from healthy migrants from Nepal, shows a negative correlation between hookworm infection and LTBI. This has been linked to the increased mycobacterial growth inhibition in the blood of hookworm-infected individuals, leading to a reduced risk of Mtb infection (37).

A protective effect of *H. polygyrus* infection in air-borne infections, such as respiratory syncytial virus (38) and *P. aeruginosa* (39), has been reported in mice. Several authors have tried to understand the impact of helminthiasis on the progression of TB using experimental models. The design of these studies should be considered to evaluate their relevance. First, most of them have been carried out in mouse strains (mainly BALB/c and C57Bl/6) that develop a sort of chronic infection based on the induction of proliferative lesions, with a strong Th1 response. Thus, they do not develop exudative lesions, characterized by neutrophilic infiltration, which are the key for developing liquefacted lesions with the capacity to become cavitated (40). So far, the only mouse strain able to develop such lesions is the C3HeB/FeJ, precisely the one used in our study (13). Also, several studies used *M. bovis* BCG as a surrogate of *M. tuberculosis* infection, thus developing a very attenuated course of infection. Thirdly, it is important to clarify which helminth species have been used. There are several studies where there is a combination of intestinal helminths, with and without a pulmonary phase in their cycle, or even filarial worms (affecting mainly the lymphatic system) thus probably reflecting different scenarios (41–44). Therefore, by changing the coinfection helminth we may have an alternative immunomodulatory effect, resulting in a new outcome than the one obtained in this experiment. The order of co-infection between nematodes and among nematodes and TB is also a determinant factor (45).

We have evaluated the parasitization with *T. muris* and *H. polygyrus*, which only affect the intestinal mucosa. We decided to use two nematodes since it is a common situation in nature (46, 47). Our results within the PLS-PM indicate that, although both species induce similar immune response (48), there was a greater effect of *T. muris* with respect to the *H. polygyrus* counterpart, evidenced in the higher weight on the helminth LV. A recent study with *M. bovis* murine coinfection showed that even when *T. muris* parasitization led to a Th2 immune background, it did not influence *M. bovis* BCG proliferation and dissemination. Coinfection increased the percentage of CD4^+^, CD4^+^IL4^+^ and CD8^+^IL4^+^ cells and decreased TNF-α secretion after BCG stimulation in the spleen when compared with the BCG infection alone. Detection of mRNA in the lung showed that coinfection decreased the expression of TGF-β, Foxp3 and IFN-γ, when compared with BCG infection alone (42). In our study, we have not been able to detect the parasitization effect on the Th2 response. However, matching the lower levels of Mtb load, these mice did present a reduction in the pro-inflammatory response in lungs. This was also observed with the oral administration of hkMm, which additionally reduced IL-17 levels, as already described (49). PLS pathway modeling supported the role of helminths and hkMm in the reduction of exudative lesions, and thus reduction of the BL. The levels of IL-10 were also lower in parasitized animals, suggesting a reduction in the regulatory response, as in Nel et al. (42). Furthermore, levels of IFN-γ did not experience significant differences between experimental groups, thus dismantling the idea of a Th2-based response in parasitization competing for the Th1 immune response. The acute-phase protein haptoglobin was reduced in sera of parasitized animals, once again pointing to an anti-inflammatory effect.

Recent estimates of hunger and food insecurity are that 821 million people are undernourished, more than 10% of the world population. This appears to be increasing in almost all regions in Africa and South America, while it is stable in most regions of Asia. A more striking fact is that the number of undernourished has been rising since 2014 (9). A low BMI is a risk factor for TB development (10), supported by several experimental models. BALB/c mice receiving 80% of the usual amount of food consumed had an impaired response to a TB vaccine (50). The administration of a low-protein-diet in Mtb infected mice increased lung BL, both in BCG-vaccinated or control animals (51). Another approach to this issue has been the study of Mtb infection in mice not expressing leptin, an adipokine reduced in malnutrition and fasting (52). This Th1 inducer increases with Mtb infection and mice lacking leptin have higher BL in advanced stages of infection (53). This was accompanied by increased numbers of PMNs in lungs. Little is known of the impact of intermittent fasting, a circumstance resembling an aspect of food insecurity. Food deprivation experiments have been carried out in mice. In our case, 48 hours deprivation induced weight reduction, which was recovered with a normal diet for five days, and an increase in oxidative stress. This regimen did not cause a reduction in albumin levels, suggesting that reduction of protection was not due to protein deprivation. Interestingly, intermittent fasting was related to a cortisol increase, probably caused by stress. Elevated levels of cortisol are linked to neutrophilia (54), which can increase neutrophilic infiltration in Mtb lesions, as has already been described in asthma patients (55). However, hkMm appears to balance this reaction as hkMm-Parasitized-Fasting animals had the same levels of cortisol as control mice.

ADA has a proinflammatory effect, by inducing Th1 response and reducing extracellular levels of adenosine (56). Parasitized-Fasting animals were the only ones that did not present lower levels of ADA than control. This matches the results in lung pathology and BL, but once again animals treated with hkMm had a distinct outcome. It appears that hkMm gave some level of protection against intermittent fasting, although it was not strong enough to be evidenced in the progression of TB in this model.

Even though the goodness of fit our PLS-PM model could be considered moderate (28, 29), it is enough to evidence the causal relationship among starvation, parasite load, environmental mycobacteria, exudative lesions (ExL), and bacillary load. Heat-killed *M. manresensis* and helminths contributed equally to diminish exudative lesions and thus bacillary load as suggested by previous research in mice (57) and cattle (58). Further investigation, however, should be conducted to elucidate the drivers of this protective effects beyond the immunological pathway used in our research.

To our knowledge, this is the first time that the impact of parasitization by intestinal helminths in the progression of active TB has been studied in an experimental model, considering important factors like food fasting and exposure to environmental mycobacteria. Our data shows an unprecedented protective impact of intestinal parasitization, which is abrogated by intermittent food fasting. This data should be considered when contemplating deworming policies and highlights the importance of good nutrition in the fight against TB.

## Data Availability Statement

The raw data supporting the conclusions of this article will be made available by the authors, without undue reservation.

## Ethics Statement

The animal study was reviewed and approved by Animal Experimentation Ethics Committee of the Hospital Universitari Germans Trias i Pujol (registered as B9900005).

## Author Contributions

Conceptualization: ES and P-JC. Data Curation: CG-A, PC, DG, LA, RV, and AT. Formal Analysis: CG-A, PC, ES, and P-JC. Funding Acquisition: P-JC. Investigation: CG-A, PC, DG, LA, RV, and AT. Methodology: ES and P-JC. Project Administration: ES and PJC. Visualization: PC. Writing – Original Draft Preparation: CG-A, PC, ES, and P-JC. Writing – Review & Editing: CG-A, PC, DG, LA, RV, AT, ES, and P-JC. All authors contributed to the article and approved the submitted version.

## Funding

The project leading to these results has received funding from la Caixa Foundation (ID 100010434), under agreement LCF/PR/GN16/10290002. This work was supported by the Plan Nacional I + D + I co-financed by ISCIII-Subdirección General de Evaluación and Fondo-EU de Desarrollo Regional (FEDER) through PC contract IFI14/00015. LA was supported by the European Commission Horizon 2020 research and innovation program under grant agreement TBVAC2020 No. 64338.

The Experimental Tuberculosis Unit is accredited by the Catalan Agency for Management of University and Research Grants (AGAUR) with code 2017 SGR500 and the IGTP is a member of the CERCA network of institutes.

ES and AT are supported by the Spanish Ministerio de Ciencia Innovación y Universidades (MICINN) through a Ramon y Cajal agreements (RYC-2016-21120; RYC-2017-22992). The research activities of CG-A are supported by a research fellow linked to the RYC-2016-21120.

## Conflict of Interest

P-JC is co-founder of two spin-offs related to tuberculosis: Archivel Farma and Manremyc.

The remaining authors declare that the research was conducted in the absence of any commercial or financial relationships that could be construed as a potential conflict of interest.
