# Supplementary material for: Protective Effect of Intestinal Helminthiasis Against Tuberculosis Progression Is Abrogated by Intermittent Food Deprivation
Source: Front Immunol. 2021 Apr 14;12:627638. doi: 10.3389/fimmu.2021.627638 (PMC8079633; doi:10.3389/fimmu.2021.627638)
Supplement: Supplementary file 3 [file Image_3.pdf]

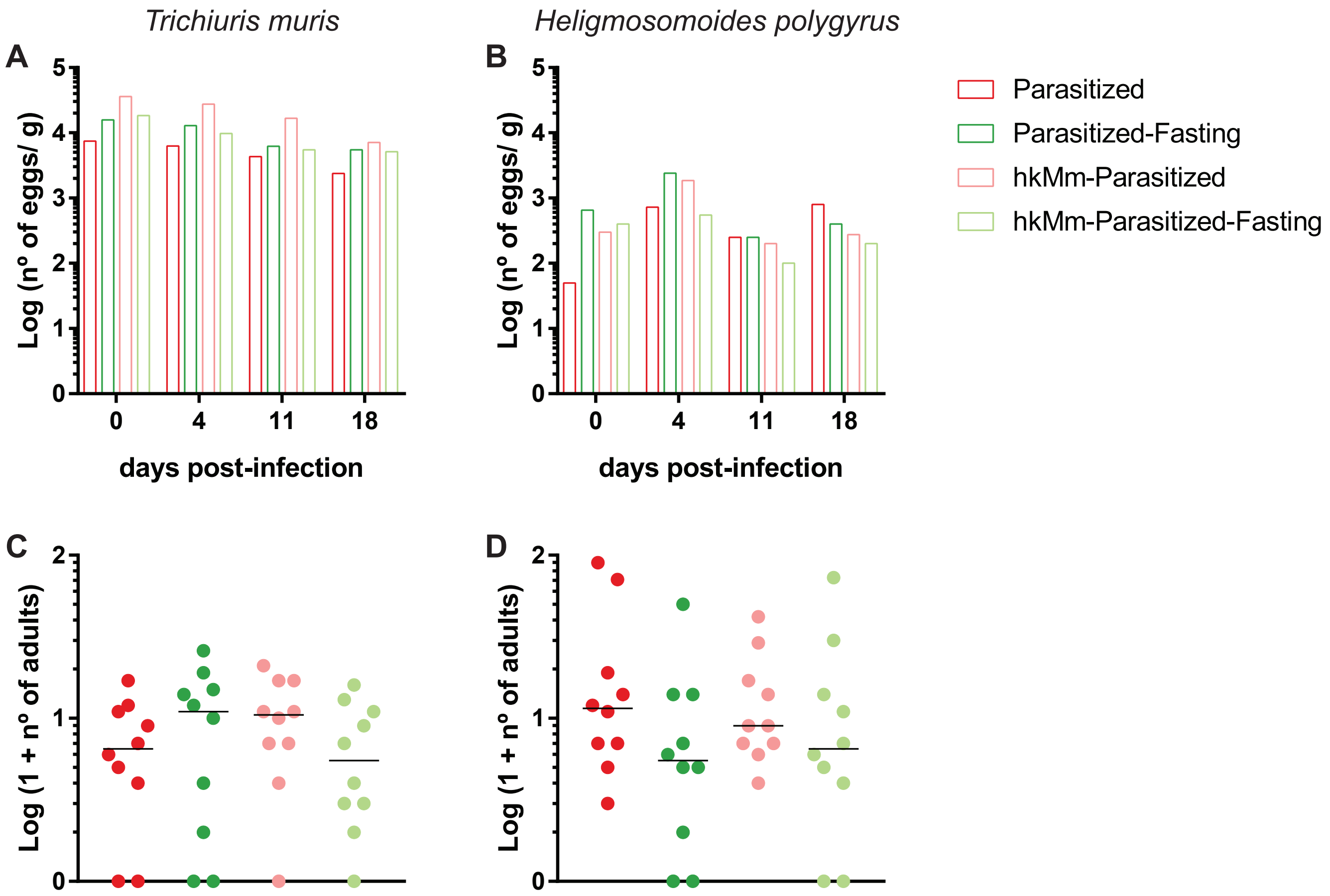

Supplementary figure 3: Recount of helminth eggs in faeces during the experiment (A-B, bars represent means of 2 cages) and adult parasites in intestines at final endpoint (C-D, each circle represents an animal and lines are medians). A and C correspond to *T. muris* and B and D are *H. polygyrus*.
