# Supplementary material for: Protective Effect of Intestinal Helminthiasis Against Tuberculosis Progression Is Abrogated by Intermittent Food Deprivation
Source: Front Immunol. 2021 Apr 14;12:627638. doi: 10.3389/fimmu.2021.627638 (PMC8079633; doi:10.3389/fimmu.2021.627638)
Supplement: Supplementary file 4 [file Image_4.pdf]

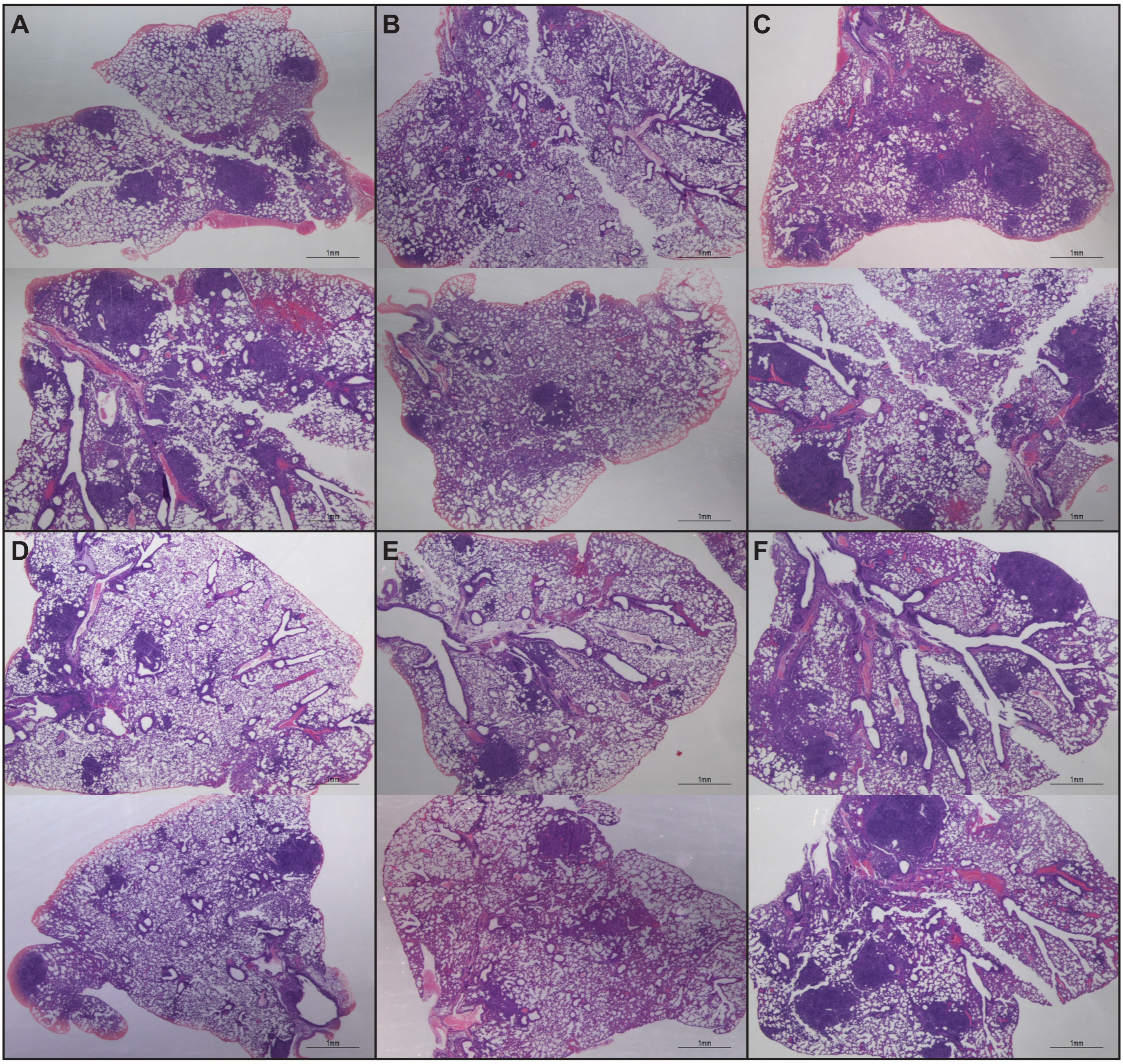

Supplementary figure 4: Representative HE stained lung sections from each experimental group at week 3 post-infection. Lines represent 1 mm. **(A)** Control, **(B)** Parasitized, **(C)** Parasitized-Fasting, **(D)** hkMm, **(E)** hkMm-Parasitized, **(F)** hkMm-Parasitized-Fasting. hkMm: heat-killed *M. manresensis*.
