# Supplementary material for: Protective Effect of Intestinal Helminthiasis Against Tuberculosis Progression Is Abrogated by Intermittent Food Deprivation
Source: Front Immunol. 2021 Apr 14;12:627638. doi: 10.3389/fimmu.2021.627638 (PMC8079633; doi:10.3389/fimmu.2021.627638)
Supplement: Supplementary file 5 [file Image_5.pdf]

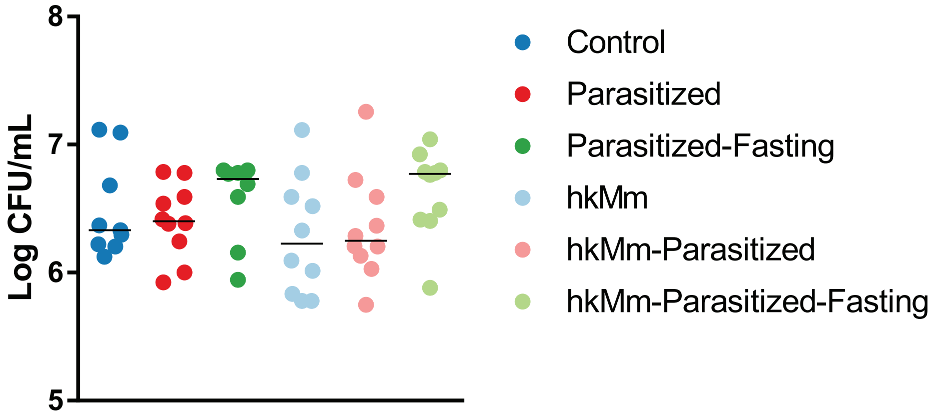

Supplementary figure 5: Spleen bacillary load at week 3 post-infection. Each circle represents an animal and lines are medians. Mann-Whitney test.
