# Supplementary material for: Protective Effect of Intestinal Helminthiasis Against Tuberculosis Progression Is Abrogated by Intermittent Food Deprivation
Source: Front Immunol. 2021 Apr 14;12:627638. doi: 10.3389/fimmu.2021.627638 (PMC8079633; doi:10.3389/fimmu.2021.627638)
Supplement: Supplementary file 8 [file Table_1.pdf]

Supplementary table 1: Description of both latent and manifest variables used for the model.

Latent variable	Manifest variable	Descriptive statistics[1]
Fasting	Categorical variable with two modalities:<br>0 = not food deprivation<br>1 = food deprivation	0.333 ± 0.475 (0 - 1)
Heat-killed *M. manresensis* (hkMm)	Categorical variable with two modalities:<br>0 = mannitol treatment<br>1 = hkMm treatment	0.5 ± 0.504 (0 - 1)
Helminths	*Heligmosomoides polygyrus*	10.00 ± 18.394 (0.00 - 89.00)
	Trichuris muris	6.367 ± 7.474 (0.00 - 30.000)
Cortisol	Cortisol	1.289 ± 0.544 (0.408 - 2.870)
Body condition	Liver weight	1.449 ± 0.274 (0.881 - 1.995)
	Kidneys weight	0.460 ± 0.153 (0.037 - 0.818)
Anti-oxidants	FRAP	0.949 ± 0.155 (0.571 - 1.353)
	TIOL	0.315 ± 0.077 (0.129 - 0.452)
	CUPRAC	0.539 ± 0.072 (0.369 - 0.719)
	TEAC2	1.083 ± 0.053 (0.946 - 1.215)
	TEAC1	0.737 ± 0.087 (0.525 - 0.943)
	PON1	10.214 ± 1.569 (7.230 - 12.930)
Oxidants	ROS	363.0 ± 78.063 (219.0 - 582.0)
	AOPP	128.64 ± 46.495 (37.80 - 293.40)
Proliferative lesions	IL-10	31.03 ± 24.357 (10.16 - 125.35)
	IL-12(p40)	21.29 ± 9.487 (4.26 - 55.42)
	IFN-γ	101.22 ± 51.037 (46.83 - 361.52)
Exudative lesions	IL-1β	202.78 ± 148.678 (27.00 - 813.98)
	IL-6	1202.9 ± 1181.824 (124.0 - 6417.7)
	TNF-α	103.94 ± 63.827 (4.98 - 331.91)
	CXCL5	302.7 ± 123.245 (107.4 - 678.0)
	IL-17	49.32 ± 57.635 (7.12 - 382.12)
	CXCL1	3252.5 ± 1986.255 (558.5 - 8580.7)
	ADA	18.25 ± 4.315 (11.78 - 28.27)
	Haptoglobin	1.921 ± 0.668 (0.430 - 3.070)
Bacillary load	Lung	14064125 ± 13072975 (2400000 - 73000000)
	Spleen	4061116 ± 3546899 (560000 - 18000000)

[1]Mean ± SD (Range).

ADA: adenosine deaminase, AOPP: advanced oxidation protein products, CUPRAC: cupric reducing antioxidant capacity, FRAP: ferric reducing antioxidant power, hkMm: heat-killed *M. manresensis*, PON1: paraoxonase 1, ROS: reactive oxygen species, TEAC: trolox equivalent antioxidant capacity.
