# Supplementary material for: Protective Effect of Intestinal Helminthiasis Against Tuberculosis Progression Is Abrogated by Intermittent Food Deprivation
Source: Front Immunol. 2021 Apr 14;12:627638. doi: 10.3389/fimmu.2021.627638 (PMC8079633; doi:10.3389/fimmu.2021.627638)
Supplement: Supplementary file 9 [file Table_2.pdf]

Supplementary table 2: Direct effects expected between latent variables.

Latent variable	Direct effects on LVs	References
Fasting	Resource limitation could affect helminths, cortisol, body condition, oxidants, antioxidants, proliferative and exudative lesions	Budischak et al., 2015 Int. J. Parasitol 45 (7): 455-463<br>Budischak et al., 2018 Front. Immunol. 8:1914<br>Budischak et al., 2018 Front. Immunol. 9:2453.<br>Beldomenico et al., 2008 Proc. R. Soc. B 275:1753-1759<br>Dang et al., 2014 PLoS ONE 9(10)<br>Rowland, 2007 CM 57(2):149-160<br>Sinha et al., 2019 J. Infect. Dis. 219:1356–63
Heat-killed *M. manresensis* (hkMm)	hkMm could affect proliferative and exudative lesions, and bacillary load	Cardona et al., 2016 Front. Microbiol. 6:1482<br>Tukvadze et al., 2016 Int J Mycobacteriol 5(S1):S101-S102
Helminths	Infection with helminths could affect body condition, cortisol, exudative and proliferative lesions, and bacillary load	Fenton, 2013 Parasitology 140: 1119–1132<br>Maizels et al., 2004 Immunol. Rev. 201:89-116<br>Mishra et al., 2014 Mucosal Immunol 7(4)<br>Mutapi, 2015 Trends Parasitol. 31(9):405-6<br>Weinstock, 2014 Clinic Rev Allerg Immunol 49:227–231
Cortisol	Cortisol could affect exudative and proliferative lesions, and bacillary load	Bongiovanni et al., 2015 Tuberculosis 95:562-569
Body condition		
Anti-oxidants	Anti-oxidants could affect oxidants and bacillary load	Lu et al., 2013 Free Radic. Biol. Med. 66:75-87<br>Mohod et al., 2011 J. Exp. Sci., 2(6): 35-37<br>Nandi et al., 2009 Immunobiology 215:443–451
Oxidants	Oxidants could affect exudative lesions	Nadeem et al., 2018 Biomed. Pharmacother.107:1196–1204
Proliferative lesions		
Exudative lesions	Exudative lesions could affect bacillary load	Cardona, 2015 Front Microbiol. 6:612
Bacillary load		
