# Supplementary material for: Protective Effect of Intestinal Helminthiasis Against Tuberculosis Progression Is Abrogated by Intermittent Food Deprivation
Source: Front Immunol. 2021 Apr 14;12:627638. doi: 10.3389/fimmu.2021.627638 (PMC8079633; doi:10.3389/fimmu.2021.627638)
Supplement: Supplementary file 10 [file Table_3.pdf]

Supplementary table 3: Results of the initial outer model before the elimination of non-significative indicators. The output shows the weight, the loading, the communality and the redundancy of each indicator of the latent variable (LV).

	weight	loading	communality	redundancy
**Fasting**				
Fasting	1.0000	1.0000	1.0000	0.000000
**hkMm**				
hkMm	1.0000	1.0000	1.0000	0.000000
**Helminths**				
Heligmosomoides polygyrus	0.0561	0.2530	0.06402	0.004839
Trichuris muris	0.9873	0.9985	0.99698	0.075362
**Cortisol**				
Cortisol	1.0000	1.000	1.000	0.301867
**Body condition**				
Liver weight	1.2393	0.9285	0.86205	0.434121
Kidneys weight	-0.4843	0.3110	0.09673	0.048711
**Anti-oxidants**				
FRAP	0.2247	0.9393	0.88237	0.418144
TIOL	0.1730	0.6540	0.42778	0.202720
CUPRA	0.2356	0.9663	0.93368	0.442460
TAC	0.1948	0.9329	0.87030	0.412423
AAH	0.2155	0.9242	0.85410	0.404748
PON	-0.2962	-0.2270	0.05152	0.024414
**Oxidants**				
ROS	1.0202	-0.9857	0.97166	0.196688
AOPP	0.1718	0.0327	0.00107	0.000216
**Proliferative lesions**				
IFNg	0.2367	0.4914	0.24147	0.037776
IL10	0.5046	0.8380	0.70223	0.109858
IL12	0.5135	0.8974	0.80529	0.125981
**Exudative lesions**				
IL.1b	0.0938	0.8369	0.70048	0.039537
IL.6	0.1633	0.8706	0.75795	0.042781
LIX	0.1605	0.6127	0.37539	0.021188
KC	0.3226	0.9190	0.84465	0.047674
TNFa	0.1574	0.8599	0.73939	0.041734
IL17	0.3698	0.6737	0.45391	0.025620
**Bacillary Load**				
Lung	1.0018	-0.9910	0.98215	0.146342
Spleen	-0.1340	0.0535	0.00286	0.000426
