# Supplementary material for: Protective Effect of Intestinal Helminthiasis Against Tuberculosis Progression Is Abrogated by Intermittent Food Deprivation
Source: Front Immunol. 2021 Apr 14;12:627638. doi: 10.3389/fimmu.2021.627638 (PMC8079633; doi:10.3389/fimmu.2021.627638)
Supplement: Supplementary file 11 [file Table_4.pdf]

Supplementary table 4: Results of the initial inner model, summarizing the relationships between each latent variable (LV) and its block of indicators. The output shows the coefficient estimates of each indicator of the LV, standard error, the t-value associated with testing the significance of the parameter listed in the first column and Pr(>|t|) that gives you the p-value for that t-test.

	Estimate	Std. Error	t-value	Pr(>|t|)
**Helminths**				
Intercept	3.98E-16	0.124	3.22E-15	1
Fasting	3.38E-01	0.124	2.74E+00	0.00817
**Cortisol**				
Intercept	2.32E-16	0.111	2.09E-15	1
Fasting	4.04E-01	0.118	3.43E+00	0.00112
Helminths	2.56E-01	0.118	2.18E+00	0.03369
**Body condition**				
Intercept	-3.90E-16	0.0972	-4.01E-15	1.00E+00
Fasting	-6.02E-01	0.1033	-5.83E+00	2.76E-07
Helminths	-1.71E-01	0.1033	-1.65E+00	1.04E-01
**Anti-oxidants**				
Intercept	-5.47E-15	0.11	-4.97E-14	1.00E+00
Fasting	5.45E-01	0.11	4.95E+00	6.71E-06
**Oxidants**				
Intercept	-2.63E-15	0.114	-2.30E-14	1
Fasting	-1.37E-01	0.136	-1.01E+00	0.31731
Anti-oxidants	-4.18E-01	0.136	-3.07E+00	0.00329
**Proliferative lesions**				
Intercept	4.05E-16	0.123	3.30E-15	1
Fasting	4.04E-01	0.144	2.81E+00	0.0069
hkMm	1.25E-01	0.129	9.67E-01	0.3377
Helminths	-2.49E-01	0.138	-1.81E+00	0.0764
Cortisol	-1.67E-01	0.153	-1.09E+00	0.2803
**Exudative lesions**				
Intercept	-8.68E-17	0.13	-6.68E-16	1
Fasting	5.90E-02	0.165	3.58E-01	0.7219
hkMm	-2.37E-01	0.138	-1.71E+00	0.0927
Helminths	-7.36E-02	0.151	-4.88E-01	0.6274
Cortisol	-1.34E-01	0.173	-7.74E-01	0.4422
Oxidants	-7.22E-02	0.151	-4.78E-01	0.6349
Proliferative lesions	1.77E-01	0.143	1.24E+00	0.2204
**Bacillary Load**				
Intercept	2.29E-15	0.117	1.96E-14	1
hkMm	-8.38E-02	0.125	-6.73E-01	0.50389
Helminths	-1.47E-01	0.13	-1.13E+00	0.26248
Cortisol	-1.55E-01	0.151	-1.03E+00	0.30963
Anti-oxidants	2.84E-01	0.14	2.03E+00	0.04737
Exudative lesions	4.02E-01	0.12	3.34E+00	0.00154
